# Restratification of survival prognosis of N1b papillary thyroid cancer by lateral lymph node ratio and largest lymph node size

**DOI:** 10.1002/cam4.1160

**Published:** 2017-08-31

**Authors:** Hye In Kim, Tae Hyuk Kim, Jun‐Ho Choe, Jung‐Han Kim, Jee Soo Kim, Young Lyun Oh, Soo Yeon Hahn, Jung Hee Shin, Hye Won Jang, Young Nam Kim, Hosu Kim, Hyeon Seon Ahn, Kyunga Kim, Sun Wook Kim, Jae Hoon Chung

**Affiliations:** ^1^ Division of Endocrinology & Metabolism Department of Medicine Thyroid Center Samsung Medical Center Sungkyunkwan University School of Medicine Seoul Korea; ^2^ Division of Breast and Endocrine Surgery Department of Surgery Samsung Medical Center Sungkyunkwan University School of Medicine Seoul Korea; ^3^ Department of Pathology Samsung Medical Center Sungkyunkwan University School of Medicine Seoul Korea; ^4^ Department of Radiology Samsung Medical Center Sungkyunkwan University School of Medicine Seoul Korea; ^5^ Department of Medical Education Sungkyunkwan University School of Medicine Seoul Korea; ^6^ Statistics and Data Center Research Institute for Future Medicine Samsung Medical Center Seoul Korea

**Keywords:** Lymph nodes, mortality, neoplasm metastasis, papillary thyroid cancer, prognosis

## Abstract

The current 7th TNM staging stratifies N1b papillary thyroid cancer (PTC) patients without distant metastasis into either stage I or stage IV merely by an age threshold (45 years). To date, no studies have adequately quantified the mortality risk of PTC patients with N1b disease. We hypothesized that incorporating lymph node (LN) factors into the staging system would better predict cancer‐specific mortality (CSM). A total of 745 nonmetastatic PTC patients with N1b disease were enrolled. We identified factors related to LNs and cut‐points using Cox regression and time‐dependent ROC analysis. New prognostic groupings were derived based on minimal hazard differences for CSM among the groups stratified by LN risk and age, and prediction of CSM was assessed. Lateral lymph node ratio (LNR) and largest LN size were significant prognostic LN factors at cut‐points of 0.3 and 3 cm. Without LN risk (lateral LNR >0.3 or largest LN size >3 cm), stage IV patients had prognosis [adjusted HR 1.10 (98% CI 0.19–6.20); *P* = 0.906] similar to stage I patients with LN risk. Patients were restratified into three prognostic groups: Group 1, <45 years without LN risk; Group 2, <45 years with LN risk or ≥45 years without LN risk; and Group 3, ≥45 with LN risk. This system had a lower log‐rank *P*‐value (<0.001 vs. 0.002) and higher *C*‐statistics (0.80 vs. 0.71) than the 7th TNM. New prognostic grouping using lateral LNR and largest LN size predicts CSM accurately and distinguishes N1b patients with different prognosis.

## Introduction

Thyroid cancer is one of the most common cancers, and its detection rate continues to rise worldwide with excellent prognosis 1, 2, 3. Unlike the other cancer AJCC/UICC staging system, the N stage of thyroid cancer is divided only by the location of metastatic lymph node (LN): N1a, central node metastasis, or N1b, lateral node metastasis. In the 7th AJCC/UICC staging system, all N1b patients 45 years or older are classified as stage IV regardless of other factors 4, 5, and risk of N1b is exaggerated. In contrast, upcoming 8th AJCC/UICC staging system 6 underestimated N1b disease by omitting that in criteria of classifying the stage, although there are a lot of evidences that survival prognosis of N1b disease is significantly worse than that of N1a 7, 8, 9. To optimize management, more tailored risk stratification of N1b patients is needed to distinguish patients with favorable survival prognosis from those with poor prognosis.

Considerable efforts have been made to find variable LN factors to subdivide papillary thyroid cancer (PTC) patients with lateral neck metastasis. Several groups have demonstrated that large LN size >3 cm is a risk factor of recurrence 10. Wang et al. suggested that LN burden >17% in the lateral neck is predictive of recurrence, but none of the evaluated LN characteristics predicted cancer‐specific mortality (CSM) 11. The number of positive LNs and extra‐nodal extension have also been suggested for LN factors related to oncological outcomes of N1b PTC patients 10. However, most studies have focused on tumor recurrence instead of CSM, and optimal cut‐points of continuous prognostic values have not been appropriately evaluated in PTC N1b patients.

The aims of this study were to assess alternative prognostic LN factors and associated cut‐points for the outcome of CSM in PTC N1b patients. We also propose an alternative prognostic system using these LN factors to stratify N1b patients more accurately.

## Materials and Methods

### Study subjects

From 1 July, 1994 to 31 December 2011, 1196 patients were diagnosed with N1b PTC disease after initial thyroid surgery at Samsung Medical Center, Seoul, Korea. Exclusion criteria included age under 18 years old at surgery (*n* = 20), distant metastasis at initial presentation (*n* = 21), less than 5 years of follow‐up (*n* = 314), recurrence within 6 months after surgery (*n* = 24), other metastatic cancer (*n* = 9), and lack of available data for LNs (*n* = 24). For accurate lymph node ratio (LNR) measurement, patients who underwent LN dissection with an inappropriate number (*n* = 39) were also excluded, based on recently proposed criteria that 6, 9, and 18 lymph nodes are sufficient for LND number with T1b, T2, and over T3 disease, respectively 12. Ultimately, a total of 745 patients were enrolled in this study. This retrospective cohort study was approved by the Institutional Review Board of Samsung Medical Center (IRB No. 2016‐09‐078) with the need for informed consent waived, and full permission was granted to review and publish information obtained from patient records.

### Study design and statistical analysis

LNs of all included cases were removed by traditional or modified radical LN dissection. No case underwent berry picking resection in which only the grossly abnormal LNs were excised 13. To identify size of metastatic LNs, we measured the longest diameter of overall (not metastatic foci) LN with metastasis using preoperative ultrasonography. RAI treatment was considered for all enrolled patient. According to the 2015 ATA guideline, the RAI dose was determined by patient's age, ETE status, size of metastatic LN, comorbidity of the patients, and preference of patients or clinicians.

The primary endpoint for survival analysis in this study was CSM. Among all mortality cases, only those recorded as code C73 (malignant neoplasm of the thyroid gland) of the International Statistical Classification of Diseases and Related Health Problems version 10 (ICD 10) for cause of death were defined as CSM. CSM‐free survival was defined as the time interval (in months) between initial surgery and death for patients with CSM and the time interval between initial surgery and the most recent follow‐up for patients without CSM 14. For additional analyses, we defined recurrence as cytopathology‐proven disease or a lesion highly suspicious for recurrence on two consecutive imaging studies [whole‐body radioactive iodine (RAI) scan, neck computed tomography (CT), or positron emission tomography (PET)‐CT or thyroid ultrasonography] with biochemically incomplete evidence [basal serum thyroglobulin (Tg) >1.0 ng/mL] 1, 14, 15.

The analysis was done in three stages. In the first stage, we evaluated the possible prognostic impact of LN factors on CSM using Cox proportional hazards analysis. Conventional clinical and pathological prognostic factors for CSM were adjusted for, such as age, sex, gross extrathyroidal extension (ETE), and therapeutic RAI (defined as a dosage of RAI 100 mCi or higher, according to the 2015 ATA guidelines) 5. In the second stage, after lateral LNR (calculated by dividing the number of metastatic LNs in lateral neck area by the total number of lateral LNs dissected) and largest LN size (defined as the longest diameter of largest LN among the metastatic cervical LNs) as continuous variable were associated with CSM, the most appropriate cut‐point combination of lateral LNR and largest LN size for predicting CSM was estimated. For this, cut‐points ranging from 0.2 to 0.5 for lateral LNR and ranging from 1 to 4 cm for largest LN size were serially matched. Each combination was analyzed by Cox proportional hazards analysis and time‐dependent ROC curve for 5 years and 10 years. We selected the optimal cut‐point combination that showed a significant *P*‐value in the Cox proportional hazard analysis and highest AUC in the time‐dependent ROC curves 16. In the last stage, to derive alternative prognostic groupings, groups with LN risk (largest size or lateral LNR over the cut‐point) and without LN risk (both largest size and lateral LNR under the cut‐point) were identified. With LN risk status and current age criteria combinations, four restratified groups were derived. Cox regression was used to calculate adjusted hazard ratios for risk of CSM in each group and alternative prognostic groupings were derived considering minimal hazard differences. The predicted performance of the alternative prognostic groupings was evaluated by comparing the *P*‐value of Kaplan–Meir log‐rank tests and *C*‐statistics 17 against current AJCC staging.

All variables, including baseline characteristics, are presented as number and percentage for categorical variables, mean ± standard deviation (SD) for continuous variables following a normal distribution, and median with interquartile range (IQR) for continuous variables not following a normal distribution. All statistical analyses were performed using IBM SPSS Statistics for Windows (Version 22.0. Armonk, NY). Significance was defined as *P* < 0.05 for two‐sided tests.

## Results

### Baseline characteristics

Table 1 shows the baseline characteristics of the 745 patients with N1b disease. Median age was 44 years (IQR 35–53 years) and most patients were female (*n* = 627, 84%). Median (IQR) largest metastatic LN size was 1.06 cm (0.80–1.57 cm) and 47 (6.3%) patients had lateral cervical LN metastasis larger than 3 cm. The median number of total metastatic and dissected LNs was 9 (5–15) and 38 (27–51), respectively. Median lateral LNR was 0.18 (0.10–0.29) and 172 (23.0%) patients had lateral LNR > 0.3. According to the 7th AJCC TNM staging system, all enrolled patients were stratified into stage I (*n* = 390, 52%) or stage IV (*n* = 355, 47%) by age criteria.

**Table 1:** Baseline characteristics of 745 N1b PTC patients

Characteristics	
Female sex	627 (84%)
Age (years), median (IQR)	44 (35–53)
AJCC staging	
Stage I	390 (52%)
Stage IV	355 (47%)
Gross extrathyroidal extension	230 (30%)
Tumor size (cm), median (IQR)	1.5 (0.9–2.2)
Total metastatic LNs, median (IQR)	9 (5–15)
Total dissected LNs, median (IQR)	38 (27–51)
Follow‐up length (month), median (IQR)	86 (74–113)
Largest LN size (cm), median (IQR)	1.06 (0.80–1.57)
Largest LN size ≥3 cm	47 (6.3%)
Lateral LNR, median (IQR)	0.18 (0.10–0.29)
Lateral LNR ≥0.3	172 (23.0%)
Therapeutic RAI	637 (85%)

PTC, papillary thyroid cancer; IQR, interquartile range; LN, lymph node; LNR, lymph node ratio; RAI, radioactive iodine.

### Identification of prognostic LN factors other than location

There were 15 cases of CSM (2%) during the median follow‐up period of 86 months. In multivariate Cox analysis (Table 2), largest LN size [adjusted HR 2.04 (95% CI 1.35–3.09), *P* = 0.001 in model 1; 1.88 (1.25–2.84), *P* = 0.002 in model 2] as well as age ≥45 [6.12 (1.25–29.84), *P* = 0.025 in model 1; 8.73 (1.83–41.56), *P* = 0.006 in model 2], and gross ETE [4.70 (1.40–15.84), *P* = 0.012 in model 1; 6.37 (1.71–23.72), *P* = 0.006 in model 2] were consistently significant prognostic factors across the different models. Neither central positive LN number nor lateral positive LN number were identified as significant prognostic factors for CSM in model 1. In contrast, lateral LNR [40.34 (3.22–504.96), *P* = 0.004] was a significant factor in model 2, which substituted the LN number of model 1 for LNR. Total LN number and ratio were not significantly associated with CSM (data not shown).

**Table 2:** Multivariate Cox proportional hazard models for cancer‐specific mortality

Variables	Model 1^a^		Model 2^b^	
	HR (95% CI)	*P*‐value	HR (95% CI)	*P*‐value
Age ≥45 years	6.12 (1.25–29.84)	0.025^a^	8.73 (1.83–1.56)	0.006^a^
Female	0.77 (0.17–3.48)	0.736	0.67 (0.14–3.06)	0.607
Therapeutic RAI	0.64 (0.16–2.55)	0.535	0.45 (0.11–1.84)	0.271
Tumor size >1 cm	0.59 (0.15–2.22)	0.440	0.45 (0.12–1.65)	0.232
Gross extrathyroidal extension	4.70 (1.40–15.84)	0.012^a^	6.37 (1.71–23.72)	0.006^a^
Largest LN size^c^	2.04 (1.35–3.09)	0.001^a^	1.88 (1.25–2.84)	0.002^a^
Central metastatic LN number^c^	0.94 (0.80–1.11)	0.481	–	–
Lateral metastatic LN number^c^	1.00 (0.91–1.11)	0.879	–	–
Central LNR^c^	–	–	0.67 (0.14–3.24)	0.623
Lateral LNR^c^	–	–	40.34 (3.22–504.96)	0.004^a^

Largest LN size—the longest diameter of largest LN among the metastatic cervical LNs; Lateral LNR–the number of metastatic LNs in lateral neck area divided by the total number of lateral LNs dissected. LN, lymph node; LNR, lymph node ratio; RAI, radioactive iodine.

a Model 1; age ≥45 years, gender, therapeutic RAI, tumor size >1 cm, gross extrathyroidal extension, largest LN size, central positive LN number, lateral positive LN number.

b Model 2; age ≥45 years, gender, therapeutic RAI, tumor size >1 cm, gross extrathyroidal extension, largest LN size, central LNR, lateral LN.

c Continuous variables.

### Cut‐point evaluation of lateral LNR and largest lymph node size

After identifying lateral LNR and largest LN size as independent prognostic LN factors, we calculated cut‐points for stratification of CSM risk. The results of Cox proportional hazard and time‐dependent ROC analyses are presented in Table 3. For predicting risk of CSM, 0.3 and 3 cm were the optimal cut‐points of lateral LNR and largest LN size, respectively, that had significant *P*‐values (*P* = 0.047, *P* = 0.021) and the highest AUC [AUC (%) time 60 months = 82.0%, 120 months = 87.74%; Table 3] among the combinations. The AUC (%) of the combination of LNR of 0.3 and largest LN size of 3 cm was much higher than that of the current AJCC TNM staging system [AUC (%) time 60 months = 72.3%, 120 months = 75.9%].

**Table 3:** Multiple Cox regression and time‐dependent ROC models for cancer‐specific mortality

Largest LN size cut‐points	Lateral LNR cut‐points	Largest LN size		Lateral LNR		AUC (%) time = 60	AUC (%) time = 120
		LN size group HR (95% HR CI)	*P*‐value	Lateral LNR group HR (95% HR CI)	*P*‐value		
1	0.2	3.84 (0.86–17.12)	0.077	1.72 (0.60–4.92)	0.309	79.10	79.31
1.5	0.2	3.31 (1.17–9.34)	0.023^a^	1.75 (0.61–4.98)	0.291	79.24	83.11
2	0.2	3.16 (1.11–8.98)	0.030^a^	1.70 (0.59–4.88)	0.319	78.44	83.28
2.5	0.2	4.81 (1.61–14.3)	0.004^a^	1.69 (0.59–4.83)	0.325	80.11	86.36
3	0.2	6.39 (2.12–19.26)	0.001^a^	1.67 (0.58–4.78)	0.335	80.21	86.01
1	0.3	3.25 (0.72–14.69)	0.124	3.37 (1.19––9.55)	0.022^a^	80.29	80.16
1.5	0.3	2.88 (1.01–8.23)	0.047^a^	3.39 (1.19–9.60)	0.021^a^	81.53	83.62
2	0.3	2.73 (0.95–7.82)	0.061	3.46 (1.21–9.85)	0.020^a^	80.72	84.03
2.5	0.3	4.11 (1.37–12.36)	0.011^a^	3.35 (1.18–9.52)	0.022^a^	81.17	87.00
**3**	**0.3**	**5.43 (1.76**–**16.70)**	**0.003** a	**3.23 (1.14**–**9.16)**	**0.026** a	**82.00**	**87.74**
1	0.4	3.52 (0.78–15.82)	0.100	2.99 (0.98–9.08)	0.052	77.84	79.69
1.5	0.4	3.20 (1.13–9.06)	0.027^a^	3.2 (1.07–9.67)	0.036^a^	76.28	78.68
2	0.4	2.96 (1.03–8.45)	0.042^a^	3.11 (1.02–9.53)	0.046^a^	75.77	80.81
2.5	0.4	4.77 (1.60–14.20)	0.005^a^	3.24 (1.08–9.69)	0.034^a^	76.84	84.23
3	0.4	6.06 (1.99–18.43)	0.001^a^	3.01 (1.00–9.02)	0.048^a^	77.41	84.76
AJCC TNM staging		–	–	–	–	72.36	75.9

LN, lymph node; LNR, lymph node ratio; HR, hazard ratio; AUC, area under the curve.

a *P* < 0.05. The combination that had significant P‐values and the highest AUC was highlighted in bold type.

### Restratification of N1b patients

Using LN risk status in addition to age criteria, N1b patients were restratified into four categories: age <45 years without LN risk, age <45 years with LN risk, age ≥45 years without LN risk, and age ≥45 years with LN risk. Compared with age <45 years with LN risk (stage I in the current AJCC TNM system), the adjusted HR of CSM for age ≥45 years without LN risk (stage IV in current AJCC TNM system) was not significantly different [1.10 (0.19–6.20), *P* = 0.906] (Table 4). A total of 269 patients (75.4%) could be down‐staged from stage IV to stage I. After these two categories with equivalent adjusted HRs were combined into one group, three alternative prognostic groups were derived: Group 1 (patients <45 years without LN risk), Group 2 (patients <45 years with LN risk or ≥45 years without LN risk), and Group 3 (patients ≥45 years with LN risk). While there was no CSM in Group 1, six (1.5%) and nine (10.4%) patients died of thyroid cancer in Group 2 and Group 3, respectively (Table 5).

**Table 4:** Restratification of N1b patients by lateral LNR and largest LN size

Variables	Cancer‐specific death		AJCC TNM staging	CSM number/total number
	HR (95% CI)	*P*‐value		
Restratification of N1b		0.002	–	–
Age <45 years without LN risk^a^	–	–	I	0/265
Age <45 years with LN risk	Ref.	–	I	2/125
Age ≥45 years without LN risk	1.10 (0.19–6.20)	0.906	IV	4/269
Age ≥45 years with LN risk	8.24 (1.73–39.24)	0.008^1^	IV	9/86
Tumor size >1 cm	0.49 (0.13–1.76)	0.277	–	–
Gross ETE	6.29 (1.81–21.86)	0.004	–	–
Female	0.66 (0.14–3.01)	0.596	–	–
Therapeutic RAI	0.50 (0.13–1.94)	0.323	–	–

ETE, extrathyroidal extension; LN, lymph node; RAI, radioactive iodine; CSM, cancer‐specific mortality.

a LN risk; lateral LNR >0.3 or largest LN size >3 cm.

**Table 5:** Alternative prognostic grouping of N1b patients

Variables	Cancer‐specific death		AJCC TNM staging	CSM number/total number
	HR (95% CI)	*P*‐value		
Alternative prognostic grouping		0.002	–	–
Group 1	–	–	I	0/265
Group 2	Ref.	–	I or IV	6/394
Group 3	7.73 (2.70–22.12)	<0.001	IV	9/86
Tumor size >1 cm	0.49 (0.13–1.78)	0.284	–	–
Gross ETE	6.45 (1.85–22.48)	0.003	–	–
Female	0.66 (0.14–2.99)	0.590	–	–
Therapeutic RAI	0.51 (0.13–1.96)	0.330	–	–

Group 1 (age < 45 years without LN risk), Group 2 (age < 45 years with LN risk or ≥45 years without LN risk), and Group 3 (age ≥ 45 years with LN risk), ETE, extrathyroidal extension; LN, lymph node; RAI, radioactive iodine; CSM, cancer‐specific mortality.

**Figure 1:**
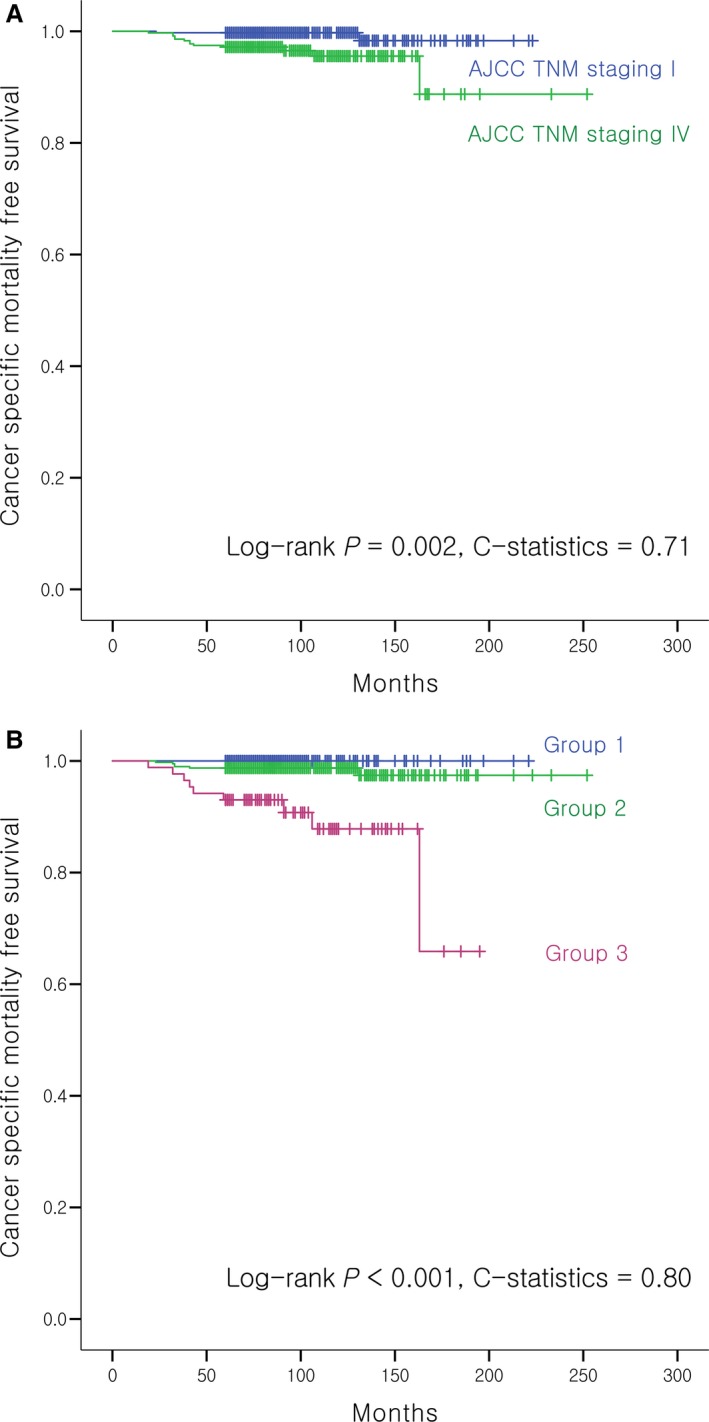
Kaplan–Meier curves for cancer‐specific mortality according to (A) the current AJCC TNM staging and (B) alternative prognostic grouping system of N1b patients.

**Figure 2:**
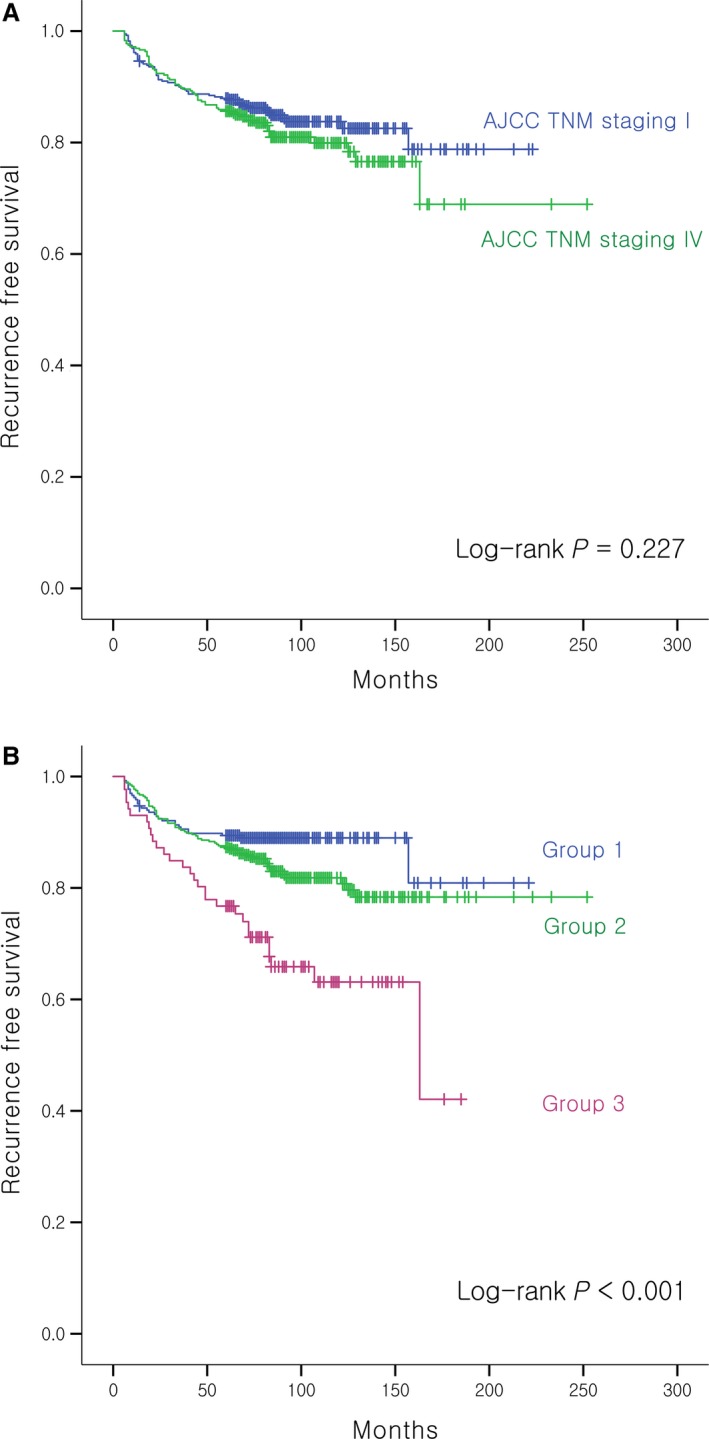
Kaplan–Meier curves for recurrence according to (A) the current AJCC TNM staging and (B) alternative prognostic grouping system of N1b patients.

The Kaplan–Meier curve of the alternative prognostic grouping system showed a lower log‐rank *P*‐value (*P* < 0.001) than that of the current AJCC TNM staging (*P* = 0.002). The *C*‐statistic for the ability of the alternative prognostic grouping system to predict risk of CSM was higher [0.80 (95% CI: 0.66–0.94)] than that of AJCC TNM staging [0.71 (0.57–0.83)], with a trend toward a significant difference between the two (*P* = 0.072) (Fig. 1). In addition, the alternative prognostic grouping also showed a significant log‐rank *P*‐value (*P* < 0.001) of the Kaplan–Meir curve for recurrence while the current AJCC TNM staging did not (*P* = 0.227) (Fig. 2).

## Discussion

In this study, lateral LNR and largest LN size had a significant impact on CSM in N1b PTC disease, with cut‐points of 0.3 for lateral LNR and 3 cm for largest LN size. The proposed alternative prognostic grouping system by lateral LNR and largest LN size had a lower *P*‐value in the log‐rank test of Kaplan–Meier curves for survival and a higher *C*‐statistic compared with the current AJCC TNM staging in N1b PTC patients.

This is the first study to identify lateral LNR as a prognostic factor for PTC. Recently, the value of LNR as a more accurate prognostic factor than LN number has been shown in other cancers such as esophageal cancer 18, gastric cancer 19, 20, colon cancer 21, head and neck cancer 22, and pancreatic cancer 23. Vincent et al. proposed LNR as an alternative to pN staging in node‐positive breast cancer 24. In contrast, the relationship between LNR and oncologic outcomes in PTC has focused only on tumor recurrence 11, 25, 26, not mortality. One study proposed total LNR as a prognostic factor of PTC using the SEER (Surveillance, Epidemiology, and End Results) dataset 27, but the authors did not adjust for the location of metastatic LNs, which is the most important criteria in N staging of thyroid cancer. Furthermore, the SEER data do not include LN dissection method, confounding accurate LNR assessment. In contrast, we knew the kind of LND that was performed for our study population and did not enroll cases with either berry picking resection or insufficient dissection. In this study, with an appropriate study population, only lateral LNR affected CSM, while all variations in LNR (total LNR, lateral LNR, and central LNR) were significant prognostic factors for recurrence. This finding suggests that the extent of the impact of LNR depends not only on how high it is but also where it is located.

As in previous studies, we found no association between the number of metastatic LNs and CSM of PTC in this study. The reason why LNR was a more accurate factor than simple number of metastatic LNs remains unclear, but it might reflect the completeness of LN dissection or potential immune responses in patients 19. Interestingly, advanced gastric cancer with strong expression of epidermal growth factor receptor (EGFR) is closely associated with high LNR, and EGFR signaling is known to affect immune response by activating regulatory T cells during human cancer development 28.

According to the 2015 American Thyroid Association guidelines, PTC patients are classified into high‐risk groups for recurrence if any metastatic LN is ≥3 cm 5. However, LN size criteria are not reflected in the AJCC 7th staging system for cancer‐specific death 4. Even though several previous studies have suggested that the largest LN size is associated with CSM 10, 29, 30, 31, controversy continues, and a size cut‐point for increasing risk of CSM has not been precisely presented before. In this study, we not only confirmed that largest LN size is an independent prognostic factor for PTC but also presented an optimal cut‐point of 3 cm using a robust statistical method. It is noteworthy that the LN size in our alternative prognostic grouping system was assessed via preoperative ultrasonography, not postoperative pathological findings, allowing patient prognosis to be predicted to some extent before surgery based on the largest LN size.

Currently, physicians treating thyroid cancers are confronted with the question of how to balance therapy so that patients with low‐risk PTC are not overtreated 1. Because N1b disease is an important risk factor for cancer‐specific death 7, 8, 9, evaluating N1a and N1b as the same prognostic group (upcoming 8th AJCC/UICC staging system 6) could underestimate risk of N1b disease. Instead, by restratification in this study, 75.7% of the stage IV cases could be down‐staged to stage I. This study does not guarantee that less aggressive treatment is safe for stage IV disease without LN risk. However, the percentage of patients treated with therapeutic RAI was not different between the two risk groups (91.9% vs. 93.3%, *P* = 0.803), suggesting that a more optimized approach should be applied to prevent overtreatment of the 75% of stage IV N1b patients who would be restratified into Group 2.

The current AJCC TNM staging system has been shown to be ineffective in predicting the recurrence of PTC. This was also evident in this study (Fig. 2A). Although the alternative prognostic grouping system was proposed to optimize prediction of mortality risk, it also qualified as a recurrence prediction tool, in contrast to the current AJCC TNM staging, suggesting that LN factors play an important role in recurrence as well as mortality.

With a relatively large study population for N1b PTC disease, this study establishes appropriate cut‐points for lateral LNR as well as largest LN size as prognostic factors. However, the retrospective study design without external validation is a limitation of the study. Although all enrolled patients underwent thyroid CT and chest X‐ray before surgery, these radiologic exams without post‐RAI whole‐body scan have possibility of missing initial distant metastasis. Therefore, the exclusion criteria of “distant metastasis at initial presentation” could not be applied for all patients strictly. However, the study result might be remained unchanged because only six patients were diagnosed with distant metastasis at 1st post‐RAI whole‐body scan. In addition, the study results were derived from the only patients with N1b disease which was a subpopulation of the DTC patients. It might not be generalizable to the all PTC population, and further study for patients without lateral cervical LN metastasis is needed.

In conclusion, N1b PTC disease is a heterogeneous group with different prognoses, and LN risk (lateral LNR and the largest LN size), in addition to patient age, was a powerful prognostic determinant for mortality outcome. By applying the proposed comprehensive alternative prognostic grouping system, physicians could prevent overtreatment of a considerable portion of N1b patients, especially those older than 45 years without LN risk.

## Conflict of Interest

All of the authors have nothing to declare.
